# Propranolol Treatment for Infantile Hemangiomas: Short-Term Adverse Effects and Follow-Up to Age Two

**DOI:** 10.1155/2019/2728952

**Published:** 2019-11-25

**Authors:** Xueqing Li, Kun Yang, Hongwen Li, Ran Huo

**Affiliations:** Department of Plastic Surgery, Shandong Provincial Hospital Affiliated to Shandong University, Jinan 250000, China

## Abstract

**Objective:**

To analyse the short-term adverse effects (AEs) of propranolol in the treatment of infantile hemangiomas (IHs) and their relevant factors, as well as the relationship between child growth and propranolol.

**Methods:**

A total of 506 patients with confirmed or suspected IHs were enrolled, and a total of 439 cases were included in the study. Short-term AEs were analysed using single-factor analysis and binary logistic regression. Out of 439 patients, 292 were enrolled to examine the effect of propranolol on 2-year-olds' height and body weight (BW), by comparison with reference range and among groups. Spearman rank correlation analysis was used to determine the relationship between BW, height, and duration of propranolol treatment.

**Results:**

Among 439 patients, 70 (16.0%) experienced AEs. Among them, 48 had gastrointestinal (GI) symptoms, 23 had central nervous system (CNS) symptoms, 8 had both symptoms above, and 7 had other symptoms. Most of the AEs occurred on the starting day (day 0), and 6 children's AEs were transient. Starting age of no older than 3 months led to more CNS symptoms, and starting age of older than 3 months was a protective factor against CNS symptoms, with an OR value of 0.303 (0.117–0.783). Height and BW of 292 two-year-old children were no less than the reference levels, although those of 3 females and 1 male were less than the average −2 standard deviation (−2SD). The height and BW of the children at the age of two was not related to the length of time of propranolol treatment.

**Conclusion:**

Oral propranolol has a good tolerance in the treatment of IHs. Oral propranolol exerts more adverse effects on the CNS of lower age children, and it has exhibited no effect on the growth of two-year-old children.

## 1. Introduction

Propranolol, a nonselective *β*-adrenoceptor antagonist, has a wide range of clinical use. As a serendipity, propranolol has become the first-line drug for the treatment of infantile hemangiomas (IHs) since 2008 [1], with higher efficacy and safety than former oral drugs, such as glucocorticoids [2, 3]. IHs need to be treated when the IH itself develops ulcer, disfigurement, obstruction, or the IH cause functional threatening [4]. Although most IHs do not require therapy, the high cure rate and safety of propranolol have made many doctors and parents dare and willing to actively treat this condition.

The general adverse effects (AEs) of propranolol include nausea, vomiting, diarrhea, and other gastrointestinal symptoms. Severe adverse events include inhibition of cardiac function, Raynaud's phenomenon, aggravating bronchospasm, hyperkalaemia, depression, somnolence, and insomnia, which can result in serious consequences [2, 4–7]. At present, there are few reports on the risk factors of AEs in infants receiving oral propranolol; moreover, the long-term side effects of propranolol in infants are unknown [8, 9].

Therefore, we performed a prospective study to document short-term adverse events in a large number of children receiving oral propranolol and analyse factors that may be associated with these adverse events; follow-up measurements of body length and weight were conducted when the children reached two years of age to elucidate the impacts of propranolol on the growth and development of young children.

## 2. Methods

### 2.1. Ethics Statement

This study was approved by the Institutional Ethics Review Board of Shandong Provincial Hospital Affiliated to Shandong University (No. 2016-308). The study was a part of a project, so the ethical number might also be used in other articles. Written informed consents were signed by the guardians of the children.

### 2.2. Participants

First, the patients were subjected to history talking, examination of the affected part and color Doppler imaging. A total of 506 consecutive outpatients with confirmed and suspected IHs at the Department of Plastic Surgery were recruited from September, 2016 to July, 2018.

*Inclusion Criteria*. (1) patients diagnosed with proliferating IHs and requiring systemic treatment (for disfigurement, obstruction, functional impairment, ulceration or proneness to ulceration, or bleeding); (2) patients suspected to have IHs, but their guardians disagreed to biopsy or watchful waiting and agreed to try oral propranolol as a diagnostic treatment; and (3) patients with normal results of routine examination, and if the children had congenital deformities, abnormal electrocardiogram (ECG) or routine blood test and thus could be treated with propranolol orally, following consultation with pediatricians. At least one of the first two criteria must be satisfied, whereas the third criterion must be met.

*Exclusion Criteria*. (1) children whose guardians refused to sign the written informed consent; (2) children who showed contraindications to oral propranolol (abnormal blood glucose, severe left ventricular dysfunction, sinus bradycardia, advanced atrioventricular block, bronchial asthma, and hepatic dysfunction); and (3) patients who needed or insisted to receive other treatments at the same time. Patients who met any of these 3 criteria were excluded.

**Figure 1:**
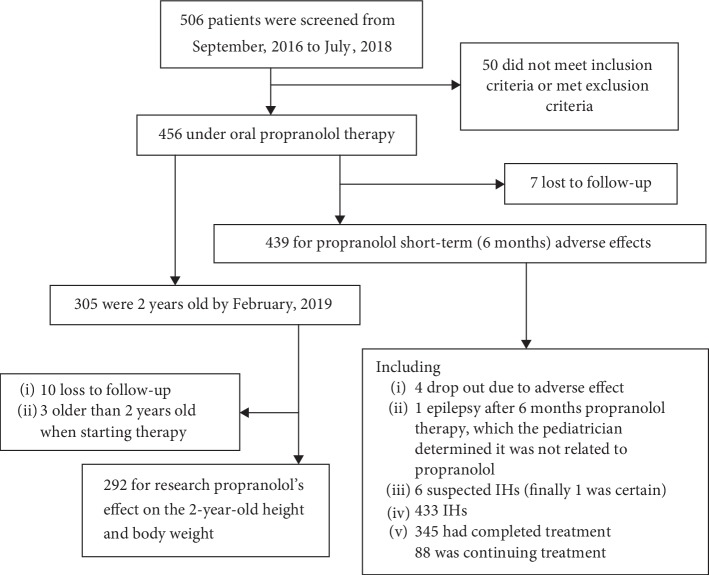
Screening and follow-up of the patients by February, 2019.

Among the screened cases, children who were 2 year old by February, 2019 were enrolled for a follow-up measurement to elucidate the effect of propranolol treatment in infants. See details in Figure 1.

### 2.3. Treatment and Follow-up

The routine examination conducted before administration of the medicine included physical examination, ECG test, routine blood test, and blood glucose test. Propranolol was administered every 12 hours during feeding or within half an hour after feeding. Dose of the starting day (day 0) was 0.5 mg/kg/day, and then the dose was gradually increased according to the reaction of the child. Generally, the dose was increased to 1 mg/kg/day on day 2 or day 3, then to 1.5 mg/kg/day on day 7, and finally to 2 mg/kg/day on day 14.

Parents could report their children's conditions to the investigators by telephone or WeChat, and routinely visit the outpatient care. The guardians were trained to recognise adverse reactions, so that they could promptly report these adverse reactions to the researchers and deal with the adverse reactions accordingly. The AEs were recorded and consulted with pediatricians when not sure if the AEs were caused by propranolol. Follow-up of short-term AEs lasted for 6 months. The height and body weight (BW) of the children were followed up by the age of 2.

### 2.4. Statistical Approach

SPSS 22.0 was used to analyse the data. Double-cross recording and checking were used to ensure the accuracy of data entry. Normality of measurement data was tested by the moment method. The data with normal distribution were expressed as mean ± standard deviation ($\overset{¯}{X}\pm S$), whereas the data with skew distribution were expressed as median and quartile (*M* (Q25, Q75)). The enumeration data were expressed as the number of cases and the percentile (*N* (%)).

Chi-squared tests, Fisher's exact test, or the Mann-Whitney *U*-test were used to analyse the 2 × 2 or row × column table. Multivariate analysis of binary logistic regression model was conducted with a variable entry criterion of 0.05 and emission standard of 0.10. The document issued by the National Health Commission of the People's Republic of China in 2009 was used as the reference standard [10]. The median of the sample was compared to the reference range using the Wilcoxon signed-rank test. Homogeneity of variance in the intragroup measurement data was tested by the *F*-test. The data with normal distribution and homogeneity of variance were used for one-factor analysis of variance (ANOVA). Otherwise, two samples were compared using the Mann-Whitney *U*-test. Spearman rank correlation analysis was used to determine the relationship between height, BW, and total duration of propranolol administration. The significance level (*α*) was set at 0.05.

## 3. Results

### 3.1. Short-Term Adverse Effects Analysis

#### 3.1.1. General Data

Out of the 506 patients screened, 439 were included in this study. Among the 439 patients, the starting age was 3.20 (1.97, 5.37) months; 301 (68.6%) patients were females; 32 (7.3%) patients were premature; 234 (53.3%) patients had IHs on the head and neck; and 70 (16.0%) patients experienced AEs. Among the patients who experienced AEs, 48 had gastrointestinal (GI) symptoms, 23 had symptoms in the central nervous system (CNS), 8 had both GI and CNS symptoms, 7 showed other symptoms, and 4 withdrew treatment owing to AEs. In children showing both GI and CNS symptoms, the AEs occurred simultaneously, except for in 1 child who had diarrhea following propranolol administration at 1 mg/kg/day and hypoprosexia following dose increase to 1.5 mg/kg/day. Therefore, it was possible that the child's mood and sleep disorders were due to factors such as diarrhea (see Tables 1 and 2). Except for 1 case of hair-thinning and 1 case of no weight gain, all other AEs occurred within 2 weeks of propranolol administration.

#### 3.1.2. GI and CNS Symptoms

Among the 48 GI cases, 39 (81.3%) occurred on day 0, whereas the remaining 9 (18.8%) occurred when propranolol was increased to 1, 1.5, or 2 mg/kg/day; among the 23 cases of CNS symptoms, 22 (95.7%) occurred on day 0 and 1 occurred when propranolol was increased to 1 mg/kg/day. There were 6 cases that had transient adverse effects, 4 of which were GI symptoms and 2 were CNS symptoms (see Table 3). The transient adverse effects mean that the symptoms disappear the next day without any treatment except continued oral propranolol according to the protocol. There were 2 cases of GI symptoms and 1 case of CNS symptoms that did not improve until completion of propranolol treatment for 6, 7, and 14 months, respectively. Except for the 6 cases of transient AEs, 3 cases of continuous AEs, and 4 cases of drop-out, the remaining improved within 6 months, and there was no child whose AE improved within 6 months due to discontinuation of propranolol at the end of treatment. For cases which improved within 6 months, median and quartile of the duration of 39 cases of GI and 17 cases of CNS symptoms were 28 (7, 56) and 28 (12, 56) days, respectively (*p* = 0.842).

Single-factor analysis showed that it could not be consider that total AEs occurrence was different in different gender, medical history, and location (*p* > 0.05), but it could be considered that patients with starting age of no older than 3 months showed more total AEs than patients with a starting age of older than 3 months (*p* = 0.007). Logistic regression based on total AEs occurrence as a dependent variable, whereas age, sex, medical history, and location as independent variables, revealed that starting age of older than 3 months was a protective factor against AEs, with an OR value of 0.508 (0.301–0.857). Statistical analysis with the same methods above showed that it could not be considered that GI symptoms were different in different starting age, gender, medical history, and location (*p* > 0.05), and no variable entered the regression model. In addition, it could be considered that patients with starting age of no older than 3 months showed more AEs in the CNS (*p* = 0.010), as determined by single-factor analysis, and starting age of older than 3 months was a protective factor against AEs in the CNS, with an OR value of 0.303 (0.117–0.783). See details in Tables 4 and 5.

#### 3.1.3. Other Symptoms

One case of lip cyanosis occurred sporadically within 10 months of propranolol treatment, but the symptom improved after termination of treatment. There were 2 cases of shortness of breath when the children got cough, but the symptoms also disappeared after treatment termination. One child showed no weight gain after 4 months of treatment (when he was 6 months old), and this state lasted for 4 months until the child finished the propranolol treatment. One case of hair-thinning occurred at 3 months after propranolol initiation; by the end of follow-up, the child had been treated for 8 months and the symptom had lasted for 5 months. Furthermore, 2 cases of cold and sweaty hands and feet occurred within 3 days of propranolol administration, which might have occurred due to hypoglycaemia because their parents administered propranolol to the children on an empty stomach; after the method of propranolol administration was corrected, the symptoms disappeared.

#### 3.1.4. Drop-Outs

**Figure 2:**
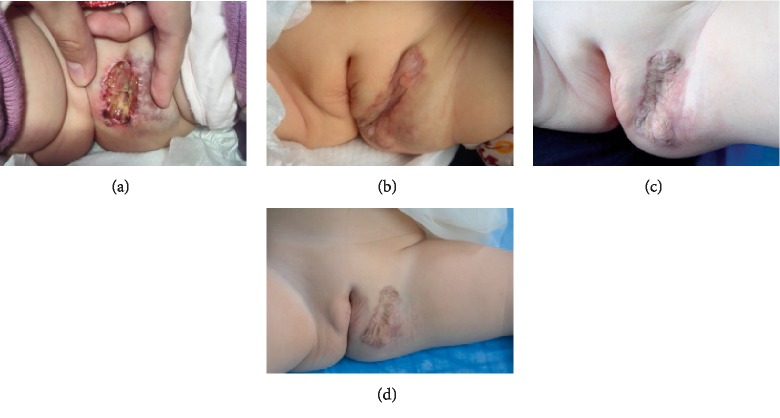
Case presentation of a 4-month-old child who had received oral propranolol for 5 days and withdrew the treatment owing to agitation and decreased total sleep time which occurred on day 0. Then the child was treated with atenolol and no AE happened. Informed consent for the publication was obtained. (a) Day 0 of treatment with propranolol. (b) 2 months after atenolol initiation. (c) 1-year old. (d) 1 year after atenolol initiation.

A total of 4 children withdrew the treatment owing to AEs, which all happened on day 0 and generally improved after treatment discontinuation; however, the guardian of one child reported that the symptom of decreased total sleep time gradually improved after 8 weeks of drug termination (see Table 2). Therefore, excluding one child whose parents refused to continue treatment, the other 3 children were subsequently treated with atenolol (see Figure 2). Among the 3 children, one showed transient sleep disorder, whereas the others showed no AE.

### 3.2. Follow-up of Propranolol Treatment in 2-Year-Old Infants and the General Data of This Cluster

Among the 439 patients under oral propranolol therapy, 305 patients were 2 year old by the end of the follow-up period, and 292 cases were qualified for data analysis. Among the 292 patients, 199 (68.2%) were females and 93 (31.8%) were males; the starting age of 82 females and 35 males was no more than 3 months; 15 females and 10 males were preterm birth; and 162 females and 76 males experienced AEs (see Table 6).

Groups were divided according to starting age, premature birth, and AEs. Some of the groups' values were higher than the reference standard, whereas others showed no statistical significance. There were 3 females and 2 males whose height and BW were more than +2 standard deviations (+2SD). In the values of less than −2SD, there were 2 females who had normal BW but lower height, and 1 female whose height and BW were lower, while 1 male showed both values less than −2SD. In brief, the height and BW of population represented by 292 two-year-old children were no less than the reference levels, whereas 9 (3.1%) were not within the average ± 2SD.

According to group comparisons, it could not be considered that the height and BW of children whose starting age were no older than 3 months were different than the children who started to take propranolol in older age. Similarly, preterm birth and AEs did not affect the average of height and BW. It could not be considered that height and BW not within ±2SD were different in different starting age, full-term or premature birth, and with or without AE (*p* > 0.05).

The height and BW of the 2-year-old children was not related to total duration of propranolol treatment. The Spearman correlation coefficient was 0.019, 0.042, −0.021, 0.015, respectively, in height of female, BW of female, height of male, and BW of male. It could not be considered that there was a positive or negative correlation between BW/height in female/male and total duration of propranolol treatment (*p* = 0.793, 0.553, 0.841, 0.887, respectively).

## 4. Discussion

Propranolol is a representative of conventional drugs that have been used to treat cardiac dysrhythmias, angina pectoris, myocardial infarction, hypertension, thyrotoxicosis, anxiety, glaucoma, alcoholism, and muscle tremor [6]. In recent years, there have been reports on propranolol as a treatment of IHs, ocular vascular proliferative diseases, acute and chronic wounds, and keloids, and as an enhancer of the therapeutic effects of radiotherapy and chemotherapy against tumours [11–17]. Development of propranolol as a treatment of IHs requires studies involving a large number of infants and young children. Due to the different pharmacokinetics and receptor sensitivity of propranolol in different races, the prescription dose of propranolol of Chinese is lower than that of Caucasians. Chinese expert consensus currently considered 2 mg/kg/d to be the recommended dose for the treatment of infantile hemangioma in China [18].

Propranolol has been used in clinical treatment for a long time and for a wide range of diseases. There are many reports on the side effects of propranolol, principally in adult volunteers; these side effects include sleep disturbances, increased dream recollection, nightmares, and diarrhea [19, 20]. With Octanol–Water Partition Coefficient (Kow) of 3.6, the lipophilic propranolol can easily cross the blood–brain barrier (BBB) [6]. Because the BBB of infants is not fully developed and oral propranolol was administered for a relatively long period in children with IHs, propranolol may have potential effects on infants [5, 21, 22].

In literature on propranolol as a treatment of IHs, AEs occurred at a frequency of 8.8–18%, with sleep problems, respiratory disorder, and asymptomatic hypotension as relatively common AEs; however, other types of AEs also occurred [23–26]. In our study, 70 (16.0%) patients experienced AEs, and the frequencies of the top 4 AEs, from high to low, were as follows: 27 (38.8%) diarrhea, 11 (15.7%) sleep problem, 5 (7.1%) increased stool frequency, and 4 (5.7%) emotion problem. There were only 2 (2.9%) respiratory problems in our study, which may occur because children with a history of neonatal pneumonia are more likely to have respiratory disorder [27]; however, all patients included in our study did not have such a history. There was no asymptomatic hypotension or bradycardia observed; because we did not measure blood pressure in the routine follow-up [28, 29], hand-held pulse oximeter was used by parents for self-test and out-patient care. Pathoglycaemia was not found in the outpatient follow-up, but as mentioned above, the 2 cases of cold and sweaty hands and feet might have occurred owing to hypoglycaemia. Although most of the AEs happened within 2 weeks of propranolol initiation, before submission, the parent of a child who had received propranolol for 10 months reported that the child suffered from hypoglycaemic coma with no obvious predisposing factors. In addition, it is noteworthy that we followed up one case of hair-thinning, which has not been previously reported in propranolol treatment for IHs [30]. On the one hand, reports of rare AE are very important so that when the phenomenon occurs, doctors can promptly reflect that it is caused by propranolol, thus allowing timely handling of the phenomenon; on the other hand, usage of propranolol as a treatment of IHs can be improved. For example, a report of dental caries suggested that sugar-free propranolol oral solutions are recommended for children [31]; propranolol may affect thermoregulation in infants, whereas atenolol may not [32].

Although some doctors previously did not advocate using propranolol as a treatment of IHs in premature infants, recent studies indicated that oral propranolol is safe for preterm and low-birth-weight infants [9, 33, 34]. It was reported that children who start oral propranolol at less than 1 month of age are more likely to develop asymptomatic bradycardia and hypotension [27]. A study also showed that younger age, premature birth, and lower BW are associated with intolerable AEs [8]. On the contrary, starting age of younger than 5 weeks dose not lead to more AEs, compared with starting age of older than 5 months [35]. Our study showed that starting age of no older than 3 months led to more AEs in the CNS, and starting age of older than 3 months was a protective factor against side effects in the CNS. In the treatment of IHs with oral propranolol, particular attention should be paid to CNS responses in younger children. Long-term follow-up after oral propranolol is currently considered safe for the physical and psychological health of infants and young children [9, 36, 37], which is consistent with our results.

In clinical setting, oral administration of montmorillonite powder and probiotics showed no obvious effect in children who showed GI symptoms following oral propranolol. Therefore, if diarrhea does not cause emotional fluctuations, dehydration, or affect sleep in children, observation treatment should be performed. With respect to sleep disorder and mood change, hydrophilic atenolol is a good alternative in cases of IHs that cannot be treated by other ways [8].

In this study, the reference BW and height values were derived from published tables, not from an untreated control group. Thus, there may be a certain discrepancy between this standard and the actual situation, especially considering improvements in nutrition levels during the past 10 years in China. Moreover, this study only followed the height and weight of children at the age of two; therefore, further evaluation of neuropsychological development in children, such as Denver Development Screening Test, Japanese SM Social Life Test, or other behavioural and psychological follow-up, are needed to reflect the long-term effects of propranolol on the CNS of children.

## 5. Conclusions

The study analysed short-term AEs of 439 children under maximum 2 mg/kg/day propranolol treatment, and the height and BW of 292 two-year-old children. A hair-thinning case was observed, which has not been reported in infants before. In the treatment of younger children with oral propranolol, especially those aged 3 months or less, special attention should be paid to their CNS symptoms. When AEs occur, the method of administration must be reconfirmed first. If the AEs are not life-threatening, observation can be taken, and the AEs which are difficult to tolerate can be replaced with atenolol. Moreover, based on our findings, propranolol may be regarded as a safe drug which does not impair the growth of children aged 2 year old or less.

## Figures and Tables

**Table 1:** Characteristics of participants.

Characteristic	Group	*n* = 439 (%)
*Age*		
	≤3 months	208 (47.4)
	>3 months	231 (52.6)
*Gender*		
	Female	301 (68.66)
	Male	138 (31.4)
*Medical history*		
	None	394 (89.8)
	Preterm birth	32 (7.3)
	Others^◆^	8 (1.8)
	Heart diseases	5 (1.1)
*Location*		
	Trunk/extremities	170 (38.7)
	Head/neck	234 (53.3)
	Perineum/female breast	35 (8.0)
*AEs*		
	No	369 (84.1)
	Yes	70 (15.9)
*GI symptom*		
	No	391 (89.1)
	Yes	48 (10.9)
*CNS symptom*		
	No	416 (94.8)
	Yes	23 (5.2)

^◆^4 cases of mild anemia, 3 cases of jaundice, 1 case of asphyxia of newborn.

**Table 2:** Adverse effects within 6-months of follow-up.

Classification	AEs	*n* = 70
Cases with only one kind of AE		
*GI symptom*		Sum = 40
	Diarrhea^▲^	27
	Increased stool frequency	5
	Active bowel sound	1
	Feeding difficulty	2
	Original diarrhea aggravation	2
	Blood-streak stool	1
	Poor appetite	1
	Vomit	1
*CNS symptom*		Sum = 15
	Agitation	3
	Hypoprosexia	1
	Reduced total sleep time, increased night awakening time^**#**^	3
	Somnolence^**#**^	2
	Somnolence, reduced sleep time at night^**#**^	1
	Reduced sleep time at night^**#**^	1
	Restless sleep^**#**^	1
	Increased night awakening time^**#**^	1
	Insomnia-early^**#**^	1
	Agitation, decreased total sleep time^**#**▼^	1

Cases with 2 kinds of AEs		Sum = 8
	Diarrhea, agitation	1
	Diarrhea, insomnia-early^**#**^	1
	Diarrhea, hypoprosexia ^◆^	1
	Active bowel sound, restless sleep^**#**^	1
	Diarrhea, decreased total sleep^**#**^	1
	Increased stool frequency; increased sleep time at night^**#**^	1
	Severe diarrhea, vomit, feeding difficulty; agitation ^▼^	1
	Severe diarrhea; decreased total sleep time^**#**▼^	1

Others		Sum = 7
	Cold and sweaty hands and feet	2
	Short of breath when the child cough	2
	Cyanosis of lips	1
	Hair thinning	1
	Growth retardation	1

Sum diarrhea cases = 34 (48.57%), sum sleep disorder cases = 16 (22.86%), ^▲^1 case of this group drop-out, ^▼^3 other drop-out cases, ^◆^had diarrhea when propranolol was increased to 1 mg/kg/day, and hypoprosexia when propranolol was increased to 1.5 mg/kg/day, other 7 children's adverse effects happened together, ^#^belong to sleep disorder.

**Table 3:** Time of adverse effects occurring and lasting.

		GI symptom	CNS symptom	*p*
Occurrence time		Sum = 48	Sum = 23	0.115^∗^
Day 0	0.5 mg/kg/day	39	22	
When propranolol was increased to				
	1 mg/kg/day	5	0	
	1.5 mg/kg/day	3	1	
	2 mg/kg/day	1	0	
Lasting time		Sum = 45	Sum = 20	0.953^∗^
Transient		4	2	
Improved before discontinuation of propranolol		39	17	
Continuous		2	1	

^∗^ Mann-Whitney *U*-test.

**Table 4:** Single factor analysis of adverse effect.

Characteristic		AE		*χ*2	*p*
		No	Yes		
		*n* (%)	*n* (%)		
*Total AE*					
Age	≤3 months	165 (44.7)	43 (61.4)	6.592	0.007^#^
	>3 months	204 (55.3)	27 (38.6)		
Gender	Female	252 (68.3)	49 (70.0)	0.08	0.782
	Male	117 (31.7)	21 (30.0)		
Medical history	No	327 (88.6)	67 (95.7)		0.366^∗^
	Preterm birth	30 (8.1)	2 (2.9)		
	Others	7 (1.9)	1 (1.4)		
	Heart disease	5 (1.4)	0 (0)		
Location	Trunk/extremities	143 (38.8)	27 (38.6)	1.651	0.438
	Head/neck	194 (52.6)	40 (57.1)		
	Perineum/female breast	32 (8.7)	3 (4.3)		

*GI symptom*					
Age	≤3 months	180 (46.0)	28 (58.3)	2.593	0.126
	>3 months	211 (54.0)	20 (41.7)		
Gender	Female	270 (69.1)	31 (64.6)	0.396	0.621
	Male	121 (30.9)	17 (35.4)		
Medical history	No	348 (89.0)	46 (95.8)		0.768^∗^
	Preterm birth	30 (7.7)	2 (4.2)		
	Others	8 (2.0)	0 (0)		
	Heart disease	5 (1.3)	0 (0)		
Location	Trunk/extremities	150 (38.4)	20 (41.7)	1.107	0.617
	Head/neck	208 (53.2)	26 (54.2)		
	Perineum/female breast	33 (8.4)	2 (4.2)		

*CNS symptom*					
Age	≤3 months	191 (45.9)	17 (73.9)	6.854	0.010^#^
	>3 months	225 (54.1)	6 (26.1)		
Gender	Female	284 (68.3)	17 (73.9)	0.322	0.651
	Male	132 (31.7)	6 (26.1)		
Medical history	No	372 (89.4)	22 (95.7)		0.329^∗^
	Preterm birth	32 (7.7)	0 (0)		
	Others	7 (1.7)	1 (4.3)		
	Heart disease	5 (1.2)	0(0)		
Location	Trunk/extremities	162 (38.9)	8 (34.8)	0.758	0.717
	Head/neck	220 (52.9)	14 (60.9)		
	Perineum/female breast	34 (8.2)	1 (4.3)		

^∗^Fisher probabilities in 2 × 2 table data, ^#^*p* < 0.05.

**Table 5:** Multivariate analysis of adverse effects.

Characteristic	B	SE	Wald	*p*	OR	95% CI
*Total AE*						
≤3 months					1	
>3 months	−0.678	0.267	6.442	0.011	0.508	0.301–0.857
Constant	−1.345	0.171	61.684	0	0.261	

*GI*						
Constant	−2.098	0.153	188.088	<0.001	0.123	

*CNS effect*						
≤3 months					1	
>3 months	−1.196	0.485	6.079	0.014	0.303	0.117–0.783
Constant	−2.424	0.253	91.785	<0.001	0.089	

Binary logistic regression model with a variable entry criterion of 0.05 and an emission standard of 0.10.

**Table 6:** Height and body weight of 2-year-old children.

Characteristic	*n* = 292 (%)	Height, cm		*n*		BW, kg		*n*	
		Average	*p* ^∗^	<−2SD	>+2SD	Average	*p* ^∗^	<−2SD	>+2SD
		$\overset{¯}{X}\pm S$/M (Q25,Q75)				$\overset{¯}{X}\pm S$			
*Gender*									
Female	199 (68.2)	87.9 ± 3.2	0.043^#^	3	3	12.00 (11.00, 12.60)	0.937	1	3
Male	93 (31.8)	98.3 ± 3.3	0.017^#^	1	2	12.50 (11.80, 13.20)	0.737	0	2

*Starting age*									
≤3 months									
Female	82 (28.1)	87.0 ± 2.8①	0.751	0	1	12.00 (11.00, 12.65)②	0.776	0	2
Male	35 (12.0)	89.0 (87.0, 90.0)③	0.514	1	1	12.50 (12.00, 12.80)④	0.183	0	1
>3 months									
Female	117 (40.1)	87.9 ± 3.5①	0.025^#^	3	2	11.85 ± 1.12②	0.891	1	1
Male	58 (19.9)	89.8 ± 3.7③	0.010^#^	0	1	12.60 (11.80, 13.50)④	0.222	0	1

*Preterm birth*									
Female	15 (5.1)	88.1 ± 3.6⑤	0.459	0	1	11.92 ± 1.44⑥	0.820	0	2
Male	10 (3.4)	87.9 ± 3.0⑦	0.527	0	0	12.17 ± 0.86⑧	0.201	0	0
*Term birth*									
Female	184 (63.0)	87.7 ± 3.2⑤	0.055	3	2	12.00 (11.00, 12.60)⑥	0.821	1	1
Male	83 (28.4)	89.5 ± 3.3⑦	0.008^#^	1	2	12.50 (12.00, 13.20)⑧	0.513	0	2

*Adverse effect*									
No									
Female	162 (55.5)	87.9 ± 3.2⑨	0.013^#^	2	2	11.94 ± 1.12⑩	0.593	0	1
Male	76 (26.0)	89.3 ± 3.2⑪	0.032^#^	1	0	12.50 (11.80, 13.20)⑫	0.768	0	1
Yes									
Female	37 (12.7)	86.9 ± 3.2⑨	0.368	1	1	12.00 (10.75, 12.00)⑩	0.462	1	2
Male	17 (5.8)	89.6 ± 3.7⑪	0.319	0	2	12.87 ± 1.61 ⑫	0.794	0	1

^∗^Wilcoxon signed-rank test: compared with standard reference, ^#^*p* < 0.05, Mann-Whitney *U*-test: ① *p* = 0.154, ② *p* = 0.704, ③ *p* = 0.192, ④ *p* = 0.109, ⑥ *p* = 0.816, ⑧ *p* = 0.281, ⑩ *p* = 0.132, ⑫ *p* = 0.968, *t*-test: ⑤ *p* = 0.648, ⑦ *p* = 0.127, ⑨ *p* = 0.106, ⑪ *p* = 0.678.

## Data Availability

The data used to support the findings of this study are available from the corresponding author upon request.
